# No tillage and residue mulching method on bacterial community diversity regulation in a black soil region of Northeastern China

**DOI:** 10.1371/journal.pone.0256970

**Published:** 2021-09-10

**Authors:** Lijun Cai, Zhenhua Guo, Jingtao Zhang, Zhijia Gai, Jingqi Liu, Qingying Meng, Xiaohu Liu

**Affiliations:** 1 Department of Land and Environment, Shenyang Agricultural University, Shenyang, Liaoning, China; 2 Jiamusi Branch of Heilongjiang Academy of Agricultural Sciences, Jiamusi, Heilongjiang, China; 3 Rice Research Institute of Heilongjiang Academy of Agricultural Sciences, Jiamusi, Heilongjiang, China; Tennessee State University, UNITED STATES

## Abstract

Soil microorganisms are important components of agricultural ecosystems; they are important in agricultural soil nutrient cycle and are easily affected by soil tillage. The response of soil microbial community to tillage is very complex, and the effect of the no tillage and residue mulching method on soil microbial diversity remains unclear. In 2019, the soil was collected from an experimental field after 10 years of continuous cultivation in the black soil area of the Sanjiang Plain in Northeastern China. In this study, the diversity and composition of the soil bacterial community and their relationship with soil properties were explored via high-throughput sequencing under no tillage with four residue mulching treatments. No tillage with 60% residue mulching (NTR3) significantly increased the alpha diversity of the rhizosphere soil bacteria and changed the composition of the bacterial community—consistent with changes in soil physicochemical properties. Proteobacteria, Acidobacteria, and Actinobacteria were the dominant phyla in the sample soil. Soil physicochemical properties explained 80.6% of the changes in soil diversity and composition, of which soil organic carbon, soil pH, and soil temperature were the principal contributors. Our results suggest that no tillage and residue mulching is conducive to increasing soil organic carbon and soil nutrient content, which is a beneficial conservation tillage measure for black soil protection in Sanjiang Plain of Northeast China. The no tillage with residue mulching, especially 60% residue mulching, alters soil bacterial community and highlights the importance of soil physicochemical properties in shaping the diversity and composition of the soil bacterial community. Our findings contribute to a broad understanding of the effects of no tillage and residue mulching on bacterial community differences and provide a scientific basis for the optimization of no tillage measures and sustainable utilization of the black soil of the Sanjiang Plain in Northeastern China.

## Introduction

Soil microorganisms are the most important active components in a soil ecosystem. About 80–90% of the biochemical reaction for soil structure maintenance, organic matter decomposition, inorganic compound transformation, nitrogen fixation and so on are mediated by microorganisms [1, 2]. Abundant microbial communities can maintain the stability of soil ecosystems [3]; however, soil microorganisms are sensitive to the soil environment, especially the changes caused by tillage—where a soil’s physicochemical properties change, leading to disturbances in the soil microbial diversity [4, 5]. Therefore, the influence of tillage practices on soil microbial diversity has attracted considerable attention [6].

No tillage and residue mulching is a farming system rapidly developed in recent years, and it has demonstrated positive effects on the ecological balance and on the societal and economic development. Straw returning has significant positive effects on soil organic carbon (SOC) fixation, mineralization, and atmospheric CO_2_ concentration regulation [7, 8]. In addition, straw returning can improve soil fertility [9], enhance soil water storage and preservation capacity [10], and adjust the characteristics of farmland microenvironment. After residue is returned, abundant carbon, nitrogen, and energy sources are provided for microorganism [11]. This creates an enriched microenvironment for microorganisms and influence the community compositions and metabolic functions of soil microorganisms further [2, 12]. Moreover, soil microorganisms act as the key factor in crop production, soil nutrient cycling, and soil sustainability [13]. Soil microorganisms are the driving force for material transformation in soil. The transformation includes ammonification, nitrification, denitrification, nitrogen fixation, cellulose decomposition, and humus decomposition and synthesis, as well as the transformation of phosphorus, sulfur, iron and other elements [14, 15]. Moreover, cellulose, hemicellulose and lignin are decompose by microorganisms into glucose, short-chain fatty acids, amino acids and CO_2_, which potentially aids agricultural crop growth [16].

As shown in most studies, no tillage can increase microbial biomass and abundance in plowed layers, especially in the topsoil [17, 18], subsequently improving the microbial diversity [19]. Plant rhizosphere—a special region formed by the intersecting of plant and soil ecosystems—is considered the second genome of plants [20, 21]. Due to the close relationship between the microorganisms in rhizosphere soil and the soil ecology [22, 23], conservation tillage has significant positive effects on the rhizosphere soil environment [24, 25]. However, considering the differences in climate, soil types, and agricultural management practices, the regulation mechanism of no tillage with regard to microbial diversity in the rhizosphere soil requires further clarification. Furthermore, most studies thus far have focused on comparing different tillage methods, whereas only a few have considered the optimization of no tillage farmland management measures.

The black soil area in Northeastern China is called the “cornerstone” to guaranteeing the national food security in China. However, this region faces excessive cultivation and predatory management of the soil [26] as well as other problems such as high land-use intensity, single planting structure, and serious soil degradation [27]. This leads to excessive consumption of farmland productivity and an imbalance between cultivation and compensation.

In recent years, as the black land conservation tillage mainly popularization technology model, no tillage and residue mulching is highly valued for the strategic protection of black soil. However, no consensus has been achieved regarding the application of residue mulching to cold regions at high altitudes [28, 29]. Although residue mulching is important for water retention and soil temperature reduction [30], it can easily result in delayed emergence and slow growth of seedlings in cold regions due to insufficient accumulated temperature [31]. In addition, most previous studies on no tillage in cold regions focused on comparing of different tillage methods, with only a few being on the management measures optimization of no tillage with residue mulching.

Here, we hypothesize that by changing the soil physicochemical conditions, no tillage and residue mulching method affects the habitat of soil microorganisms and alters the compositions and potential functions of the microbial communities in the rhizosphere soil. Therefore, in this 10-year-long experiment, the effects of no tillage and residue mulching on the soil physicochemical properties and bacterial diversity were analyzed through high-throughput sequencing.

Therefore, the purpose of this study was to

1. Compare the differences of the bacterial communities in rhizosphere soil through different residue mulching methods under no tillage;
2. Explicitly state the way residue mulching affects soil physicochemical properties and bacterial community diversity in rhizosphere soil further; and
3. Ascertain the best residue mulching method suitable for no tillage in the black soil area of Northeastern China and optimize no tillage cultivation technology for cold regions.

## Materials and methods

### Study site

This experiment was conducted in the experimental field of Jiamusi Branch of Heilongjiang Academy of Agricultural Sciences (45°56′N–48°28′N, 129°29′E–135°5′E) in Northeastern China from 2010 to 2020. This region has a continental monsoon climate in the middle temperate zone. Here, the winter is long, and the summer is short; the annual average temperature is 3°C, annual average precipitation is 530 mm, the sunshine duration is 2525 h, the effective accumulated temperature is 2590°C, and the frost-free period is about 130 days. The altitude of the test site is 53 m, and the test soil was meadow soil with a 30-cm-thick layer of black soil. The basic properties of the 0–20 cm of soil layer are as follows: soil organic matter, 28.07 g·kg^-1^; total nitrogen (TN), 1.40 g·kg^-1^; total phosphorus (TP), 0.87g·kg^-1^; total potassium (TK), 19.59 g·kg^-1^; alkali-hydrolyzed nitrogen, 110.11 mg·kg^-1^; available phosphorus, 56.24 mg·kg^-1^; and available potassium, 172.83 mg·kg^-1^_._

### Experimental design

A randomized complete block design was adopted in this experiment, and soybean–maize rotation was used as the cropping system. The experimental plot area was 24.3 m^2^ (6 × 4.05 m^2^). The field microplots were built in October 2009; 1-m-deep grooves were dug around each microplot and blocked with linoleum papers. The interspaces between them were filled with soil to ensure the independence of the microplot. In total, four treatments were set: no tillage with all crop residues removed at harvest (NTR1), no tillage and 30% crop residues chopped and spread evenly on the soil surface (NTR2), no tillage and 60% crop residues chopped and spread evenly on the soil surface (NTR3), and no tillage and all crop residues chopped and spread evenly on the soil surface (NTR4). Each treatment was repeated three times. Crop residues were treated as follows: all residue in the field was collected after annual crop harvest; it was dried and then cut into 10-cm-long pieces. These pieces were then sprinkled onto the soil surface evenly.

For 100% residue mulching treatment, 12,000 kg·hm^-2^ maize residue (dry matter) was used; moreover, residue consumption was converted according to this standard for other treatments. Cropping pattern was conventional planting, where seeds were sown in artificial furrows. The row spacing was 45 and 65 cm in soybean and maize, respectively, whereas the basic seedling number of soybean and maize was 75,000 and 335,000 plants per hectare, respectively. Fertilizer composition for soybean and maize crops were as follows: pure N, 60 kg·hm^-2^; pure P_2_O_5_, 120 kg·hm^-2^; and pure K_2_O, 80 kg·hm^-2^. Moreover, urea (138 kg·hm^-2^) was used as top-dressed nitrogen for maize at the 7-leaf stage. Soil closed chemical weeding and no tillage management was applied during growth periods. In addition, the cropping system was set as harvesting once a year.

### Soil sampling and soil property analysis

Soil samples were collected in August 2019 after 10 years of continuous cultivation. In brief, we randomly selected maize crops with a similar growth at the V12 period and shook off big clots to brush and collect the rhizosphere soil that was tightly attached to the root surface [32]. The collected samples were then stored at −80°C until the soil bacterial diversity was determined. In addition, after the soil around the rhizosphere was collected and mixed, about 500 g of the soil was separately collected and stored 4°C until microbial biomass and soil chemical properties were determined using the method of quartering after air drying. These experiments were repeated three times per experimental plot.

Soil temperature was determined on an automatic temperature recorder (TR-71U, Japan). Soil moisture content was measured after oven drying as follows. Soil pH was measured in a mixture of soil and deionized water at 1:2.5 (w/v) by using a pH meter (SevenCompact S220, Shanghai, China). A routine method was used to determine soil bulk density (BD) [33]. Moreover, SOC and soil TN were determined using the Walkley–Blakck [34] and Kjeldahl [35] methods. For soil TP and TK, soil was first digested using HF-HNO_3_ and then analyzed through molybdate colorimetric measurement and flame photometry, respectively [36]. Soil microbial biomass was elucidated through chloroform fumigation and K_2_SO_4_ extraction [37].

### DNA isolation and Polymerase Chain Reaction (PCR) conditions

Total soil microbial DNA was extracted from four soil samples using the E.Z.N.A. Soil DNA Kit (Omega Bio-Tek, Norcross, GA, USA), according to the manufacturer’s protocol. Each sample testing was repeated three times, and about 0.5 g of the soil was used for each repetition. The extracted DNA was analyzed on Nanodrop-2000 (Thermo Fisher Scientific, Wilmington, DE, USA) and stored at −20°C.

With purified DNA as template and the set of general primers (338F: 5′-ACTCCTACGGGAGGCAGCA-3′ and 806R: 5′-GGACTACHVGGGTWTCTAAT-3′), PCR amplifications were performed on the V3–V4 highly variable region of bacterial 16S RNA gene.

The PCR reaction system included the DNA template (2 μL), Q5 reaction buffer (5×, 5μL), Q5 High-Fidelity GC buffer (5×, 5 μL), forward and reverse primer (10 μM, 1 μL each), Q5 High-Fidelity DNA Polymerase (5 U/μL, 0.25 μL), and dNTPs (2.5 mM, 2 μL), all diluted to 25 μL using ddH_2_O.

Moreover, the PCR procedure was performed as follows: pre-denaturation for 2 min at 98°C, followed by 25 cycles of denaturation for 15 s at 98°C, annealing for 30 s at 55°C, and extension for 30s at 72°C and then a final extension for 5 min at 72°C. The target fragment of PCR product was recovered using a DNA recovery kit (Tiangen) and was quantitatively detected based on the fluorescence quantitation (SystemQuantif-Luor-ST). According to the requirements of library construction, the samples were mixed at equal proportions and sent to Beijing Biomarker Technologies (Beijing, China) for sequencing on a HiSeq 2500 PE2500 (Illumina, USA).

### Date processing and bioinformatics analysis

The paired-end reads obtained through sequencing were spliced using FLASH (version 1.2.7, http://ccb.jhu.edu/software/FLASH/)) [38] and filtered using Trimmomatic (http://www.usadellab.org/cms/?page=trimmomatic) [39]. Next, chimeras were removed using UCHIME (version 8.1, http://drive5.com/uchime) to obtain high-quality Tag sequences. Based on USEARCH (version 10.0), operational taxonomic units (OTUs) were filtered with 0.005% of the number of sequenced reads as the threshold. The OTUs were clustered at the level of 97% similarity. Taxonomy was assigned to all OTUs by searching against the Silva databases (Release128, http://www.arb-silva.de) using uclust within QIIME (version 1.8.0). Mothur (version v.1.30, http://www.mothur.org/) was used to analyze alpha diversity, including Chao1, ACE, and Shannon and Simpson diversity indexes. Beta analysis was performed on QIIME (version 1.9.1), whereas PCoA was performed based on the UniFrac algorithm and using R (version 2.15.3). SPSS (version 21.0; SPSS Inc., Chicago, IL, USA) was used for univariate analysis of variance (i.e., one-way ANOVA), with the significant level set at 0.05.

## Results

### Soil physicochemical properties

After 10 years of continuous no tillage mulching, the soil physicochemical properties of each treated microplot changed significantly (Table 1). Soil temperature, soil pH, SOC, and soil TN were significantly different among different treatments (P < 0.05). With an increase in straw mulch amounts, soil pH and temperature demonstrated a decrease. By contrast, SOC and soil TN were higher after NTR3 and NTR4 than after NTR1. However, no significant difference was found with regard to soil moisture content, BD, and TP (P > 0.05).

**Table 1:** Effects of no tillage and residue mulching treatments on soil physiochemical properties.

Treatment	Moisture (%)	BD (g cm^3^)	Temp (°C)	pH	SOC (g·kg^-1^)	TN (g·kg^-1^)	TP (g·kg^-1^)	TK (g·kg^-1^)
NTR1	18.62±0.60 a	1.31±0.05 a	24.92±0.07 a	6.26±0.01a	13.98±0.09 c	1.28±0.01 b	0.93±0.01 a	19.41±0.08 c
NTR2	19.17±1.09 a	1.42±0.06 a	24.35±0.02 b	6.17±0.04 a	14.53±0.05 b	1.36±0.02 a	0.92±0.02 a	19.98±0.04 a
NTR3	20.86±0.13 a	1.47±0.09 a	24.04±0.07 c	5.91±0.05 b	16.84±0.14 a	1.43±0.02 a	0.90±0.01 a	19.77±0.02 b
NTR4	20.23±0.46 a	1.51±0.02 a	23.74±0.02 d	5.83±0.06 b	17.12±0.03 a	1.41±0.04 a	0.92±0.01 a	19.45±0.07 c

BD, soil bulk density; SOC, soil organic carbon; TN, total nitrogen; TP, total phosphorus; TK, total potassium; NTR1, no tillage and all crop residue removed; NTR2, no tillage and 30% crop residue mulching; NTR3, no tillage and 60% crop residue mulching; NTR4, no tillage and all crop residue mulching. Values are shown as means ± SD of three biological replicates. Different letters indicate significant differences (ANOVA, P < 0.05, Tukey’s HSD post-hoc analysis).

### Soil microbial biomass

Significant differences were detected among the treatments in soil microbial biomass carbon (MBC), microbial biomass nitrogen (MBN), and microbial biomass phosphorus (MBP) content (P < 0.05; Table 2). Soil MBC and MBN contents after NTR3 were the highest, and they were higher than those after NTR1 and NTR2. Soil MBP content ranged from 0.78 to 1.42 mg·kg^-1^, which were highest in NTR4. The microbial entropy after NTR2, NTR3, and NTR4 was significantly higher than that after NTR1; moreover, soil MBC and MBN after NTR3 was the highest among the treatments.

**Table 2:** Effects of no tillage and residue mulching treatments on soil microbial biomass.

Treatment	MBC (mg·kg^-1^)	MBN (mg·kg^-1^)	MBP (mg·kg^-1^)	MQ (%)	MB C/N
NTR1	179.30±5.33c	24.04±2.24d	0.78±0.02d	1.28b	6.57bc
NTR2	205.48±7.92b	29.82±2.31c	0.95±0.11c	1.41a	6.89b
NTR3	248.63±2.89a	36.52±1.09a	1.23±0.07b	1.48a	7.71a
NTR4	239.47±4.97a	33.36±1.76b	1.42±0.04a	1.40a	6.97b

MBC, microbial biomass C; MBN, microbial biomass N; MBP, microbial biomass P; MQ, microbial quotient; MB C/N, microbial biomass C/N. NTR1, no tillage and all crop residue removed; NTR2, no tillage and 30% crop residue mulching; NTR3, no tillage and 60% crop residue mulching; NTR4, no tillage and all crop residue mulching. Values are shown as means ± SD of three biological replicates. Different letters indicate significant differences (ANOVA, P < 0.05, Tukey’s HSD post-hoc analysis).

### Soil bacterial community diversity

The bacterial diversity sequencing was performed on an Illumina HiSeq sequencing platform. In total, 854,576 reads were generated in 12 samples after sequencing, and 820,664 clean tags were generated after double-ended reads were spliced and filtered (S1 Table). Through clustering operation, bioinformatic statistical analysis was performed on the 97% OTU similarity, where 1438 OTUs were generated from 12 samples (ranging from 1286 to 1400). According to the RDP classification of all OTUs, the bacterial groups can be divided into 20 phyla, 66 classes, 140 orders, 221 families, 391 genera, and 432 species (S2 Table).

**Fig 1:**
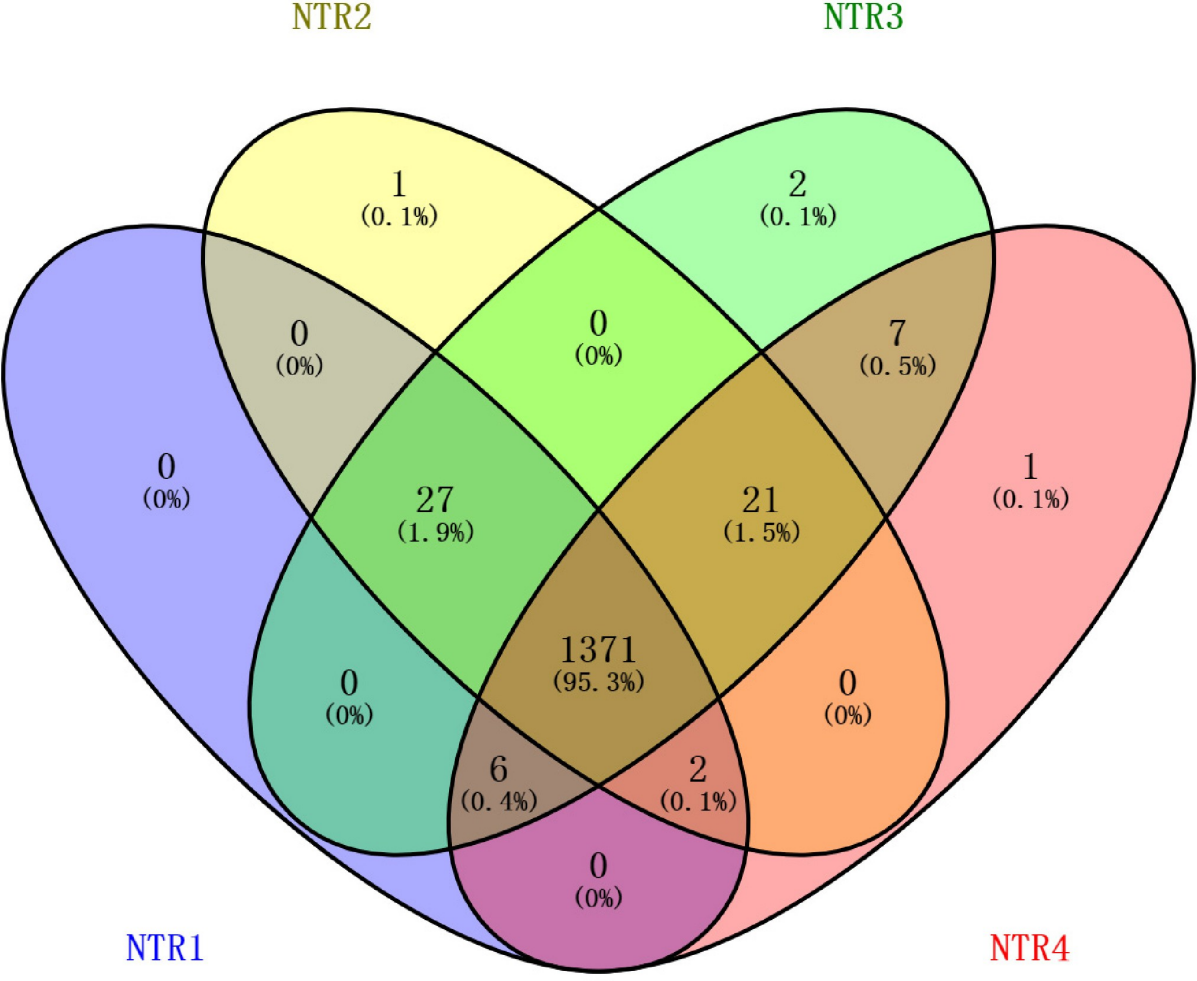
Venn diagrams showing the shared and unique OTUs after different no tillage and crop residue mulching treatments. NTR1, no tillage and all crop residue removed; NTR2, no tillage and 30% crop residue mulching; NTR3, no tillage and 60% crop residue mulching; NTR4, no tillage and all crop residue mulching.

The Good’ s coverage index of each sample was >0.996, and the precipitation curves were close to the saturated phase, indicating that the sequencing depth of each sample reached a sufficient level, which could accurately reflect the overall microbial community libraries of soil bacteria. For all treatments, the distribution order of all OTUs was as follows: NTR3 > NTR2 > NTR1 > NTR4; here, more OTUs commonly existed in NTR3 and NTR4 (Fig 1).

**Fig 2:**
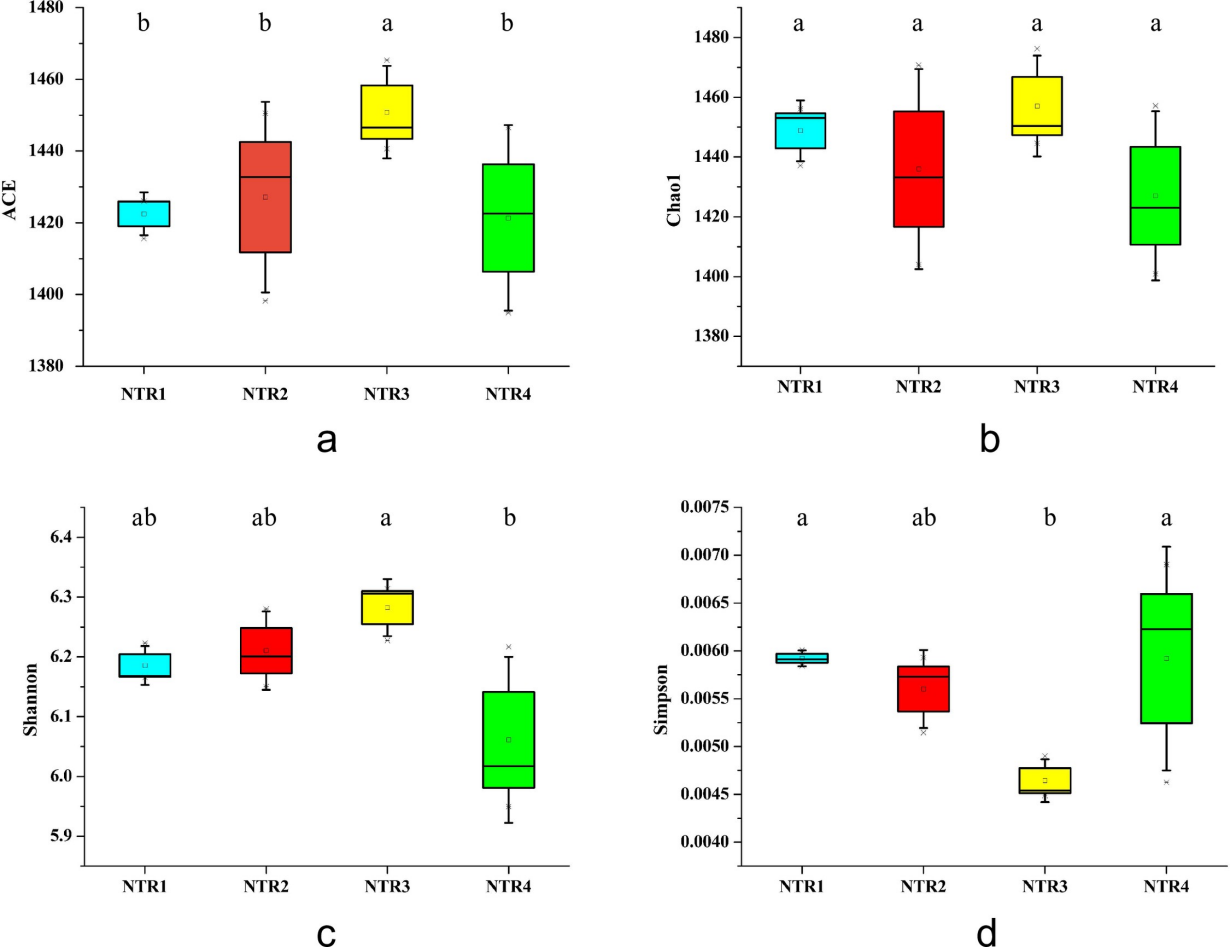
The diversity and richness of soil bacterial according to the Chao1 (a), ACE (b), Shannon (c), and Simpson (d) indexes after different no tillage and crop residue mulching treatments. NTR1, no tillage and all crop residue removed; NTR2, no tillage and 30% crop residue mulching; NTR3, no tillage and 60% crop residue mulching; NTR4, no tillage and all crop residue mulching. Boxplots with different letters above the boxes denote significantly different means (P < 0.05).

Furthermore, the numbers and diversities of microorganism species contained in samples were represented through alpha diversity analysis. As shown in Fig 2 and S3 Table, the distribution order of the ACE and Chao1 index richness for all the treatments was as follows: NTR3 > NTR2 > NTR1 > NTR4 and NTR3 > NTR1 > NTR2 > NTR4, respectively. Moreover, the Shannon index was significantly higher after NTR3, NTR2, and NTR1 than after NTR4, whereas the Simpson indexes was significantly after NTR4 and NTR1 than after NTR3. Thus, NTR3 was beneficial to the enhancement of the bacterial richness and diversity of the rhizosphere soil.

### Analysis of beta diversity and bacterial community composition

**Fig 3:**
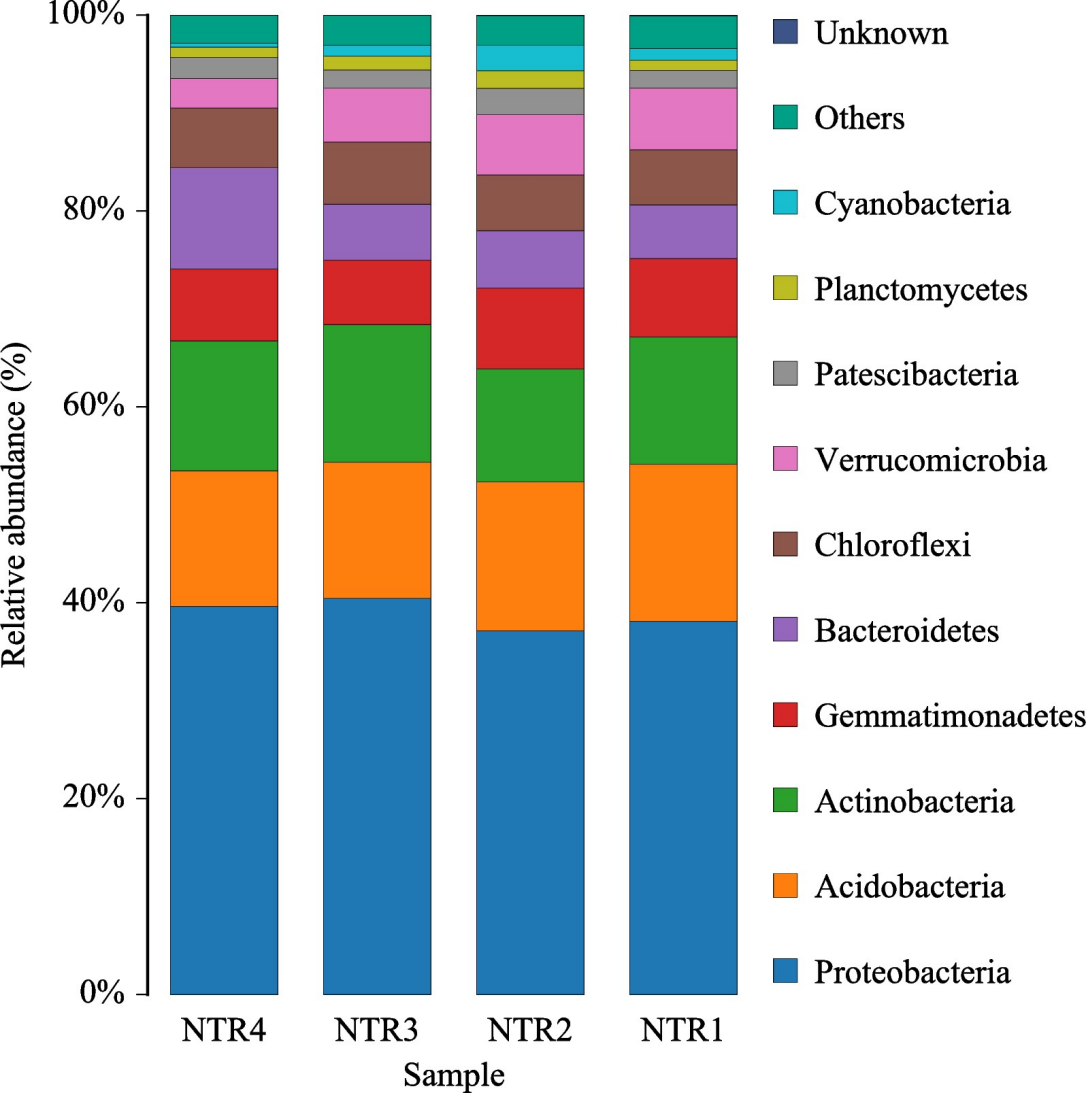
Relative abundance of top 10 soil bacterial phyla for all samples after different no tillage and crop residue mulching treatments. NTR1, no tillage and all crop residue removed; NTR2, no tillage and 30% crop residue mulching; NTR3, no tillage and 60% crop residue mulching; NTR4, no tillage and all crop residue mulching. The stacked bar graph represents relative abundance of the major phyla.

On the basis of the comparisons of the taxonomic diversity of bacterial communities among the samples at the phylum level, the top 10 dominant bacterial phyla were Proteobacteria (37.17%–40.29%), Acidobacteria (13.91%–16.15%), Actinobacteria (11.77%–14.37%), Gemmatimonadetes (6.64%–8.40%), Bacteroidetes (5.48%–10.18%), Chloroflexi (5.50%–6.25%), Verrucomicrobia (2.91%–6.15%), Patescibacteria (1.77%–2.52%), Planctomycetes (0.53%–2.39%), and Cyanobacteria (0.40%–2.59%)—accounting for 95.89%–97.07% of the relative abundance of bacterial communities (Fig 3; S4 Table). As shown in the above results, the bacterial communities at the phylum level were similar among the soil samples; however, the relative abundances of the bacterial communities differed significantly within each other, especially after NTR3 and NTR4—where the relative abundance of the phyla Proteobacteria, Actinobacteria, Bacteroidetes, and Chloroflexi increased and that of Acidobacteria, Gemmatimonadetes, and Verrucomicrobia decreased. In addition, the relative abundance of the phyla Patescibacteria, Planctomycetes, and Cyanobacteria was increased after NTR2 (P < 0.05).

**Fig 4:**
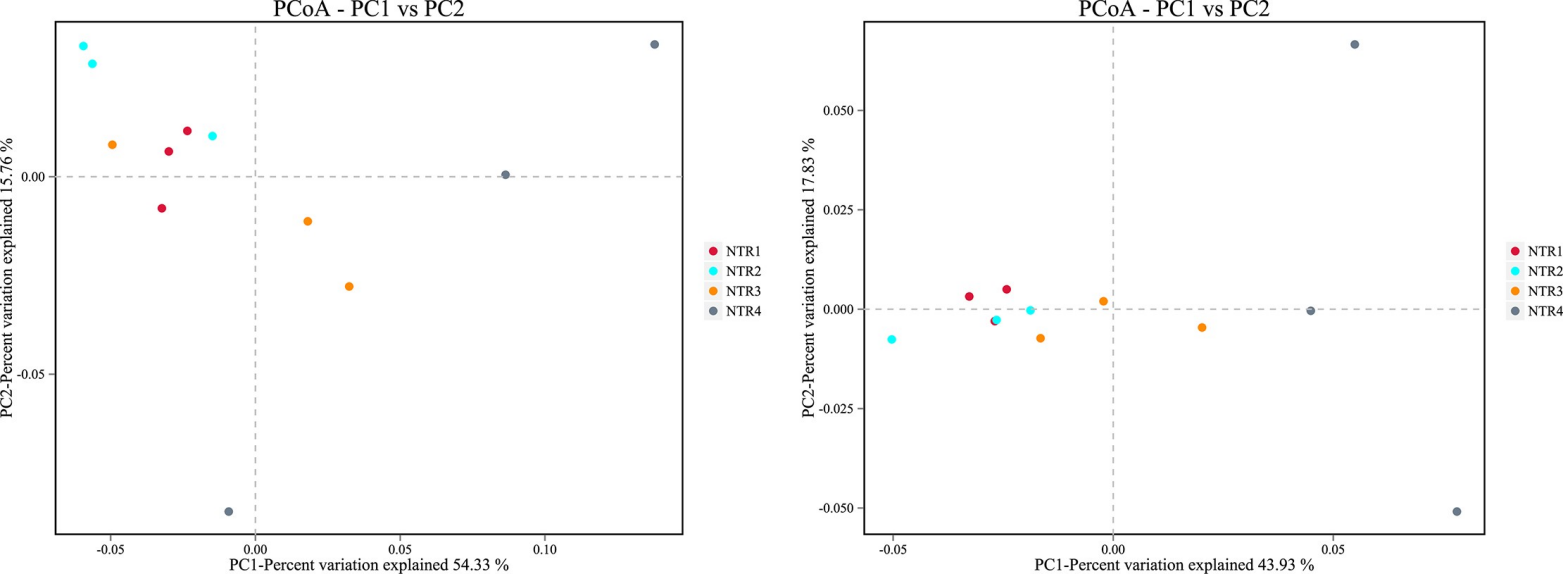
Summary of principal coordinate analysis of soil bacterial composition after different no tillage and crop residue mulching treatments based on the (a) weighted and (b) unweighted UniFrac distances. NTR1, no tillage and all crop residue removed; NTR2, no tillage and 30% crop residue mulching; NTR3, no tillage and 60% crop residue mulching; NTR4, no tillage and all crop residue mulching.

The PCoA results demonstrated that there were differences in bacterial community structure among the soil samples. In the unweighted and weighted UniFrac distance analysis for bacterial community structure, PCo1 explained 54.33% and 43.93% of the total variance, respectively, and PCo2 explained 15.76% and 17.83% of the total variance, respectively (Fig 4A and 4B). The spatial distance of soil samples treated using NTR1 and NTR2 was relatively small, whereas the spatial separation between NTR3 and NTR4 was highly obvious; this result indicated that bacterial community structure of rhizosphere soil microdomain in both the NTR3 and NTR4 treatments were significantly different from those in NTR1.

### Relationship between soil physicochemical properties and soil bacterial community structure

**Fig 5:**
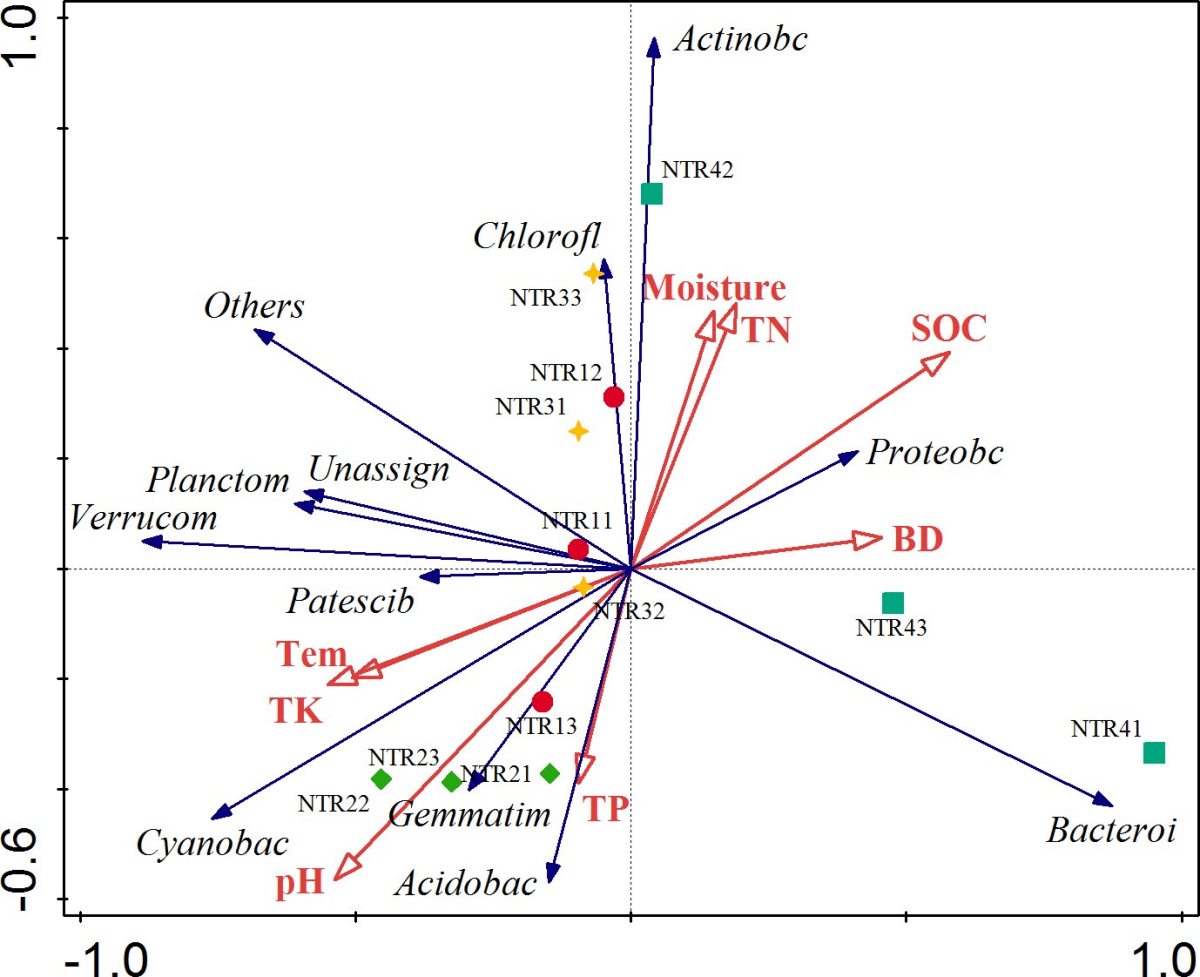
Summary of redundancy analysis, showing the relationships between soil parameters and soil bacterial community structure. Red lines represent soil parameters, blue lines represent the bacterial phylum-level taxonomy, and graphics of different colors represent soil samples from all replicates (n = 3) of each crop residue mulching treatment. Numbers after treatment abbreviations denote the experimental replication. BD, soil bulk density; SOC, soil organic carbon; TN, total nitrogen; TP, total phosphorus; TK, total potassium; NTR1, no tillage and all crop residue removed; NTR2, no tillage and 30% crop residue mulching; NTR3, no tillage and 60% crop residue mulching; NTR4, no tillage and all crop residue mulching.

Furthermore, redundancy analysis was performed to determine the relationship between soil physicochemical properties and soil bacterial community diversity. As shown in Fig 5, axes 1 and 2 explained 52.29% and 16.48% of the total variance in soil bacterial community structure, respectively (S5 Table). Of the bacterial community compositions, Proteobacteria and Actinobacteria were significantly positively correlated with SOC, soil TN, and soil moisture content; Gemmatimonadetes, Cyanobacteria, and Acidobacteria were significantly positively correlated with soil pH, TP, TK, and temperature; and Patescibacteria and Verrucobacteria were significantly positively correlated with soil temperature and TK. In addition, our testing results indicated that soil SOC (P = 0.068), soil pH (P = 0.081), and soil temperature (P = 0.098) were the three most important contributors to bacterial community variation (S6 Table). Moreover, the degree of the contribution of soil physicochemical properties to soil bacterial community variance was in the following order: SOC > soil pH> soil temperature> soil TK > soil BD > soil TN > soil moisture> soil TP.

## Discussion

### Effects of on soil properties

In the present study, different residue mulching methods in a soybean–maize rotation system over 10 consecutive years were found to have varied effects on the soil physicochemical and biological properties. Although soil temperature, SOC, soil pH, soil TN, and soil TK varied significantly among treatments, the largest differences were noted in soil temperature and SOC (Table 1).

Previous studies have shown that residue mulching can not only increase SOC retention by reducing the fluctuation of surface soil temperature and water content but also reduce the erosion of SOC by reducing surface runoff [8, 10]. However, whether SOC synchronously increases with an increase in residue returning quantity remains controversial. Many scholars have reported that with as the amount of straw returning increases, SOC content and carbon pool activity increases and positively correlates with the amount of residue returning and the number of years of returning [40, 41]; this result is consistent with the current results. However, Bai et al. [42] found that residue mulching did not have a superposition effect on the improvement of SOC in interannual paddy–upland rotation farmland and that SOC did not increase synchronously with an increase in residue returning amount because excessive residue mulching reduced soil pH and decomposition rate of straw—all of which was not conducive to SOC accumulation.

The input of exogenous organic matter can accelerate the microbial decomposition rate of organic carbon sources, ensure high microbial biomass content, and strengthen the assimilation and fixation of microorganisms [43]. Because long-term continuous no tillage and residue mulching does not disturb the soil, and plant residues accumulate on the soil surface in successive years; this cause considerable changes in soil biological characteristics [44] In the current study, soil MBC, MBN, and MBP contents significantly increased under no tillage and residue mulching (Table 2). The reason for this difference is that soil microorganisms are mainly heterotrophic populations, and their life activities involve consuming a certain amount of energy. Treatment with a large amount of residue mulch provides sufficient energy for microorganisms to maintain life activities.

Some studies have also suggested that too high an amount of residue returning can adversely impact the metabolic activity of soil microbial carbon sources [45], which may be related to factors such as the soil type, soil fertility, and climatic conditions of the test site. Due to the high organic matter content of black soil in Northeastern China, excessive residue returning can lead to an imbalance in soil carbon and nitrogen ratio, resulting in competition between microorganisms and plants for nitrogen. This can thus inhibit microbial metabolic activity. Therefore, in this study, when the amounts of residue returning increased from 60% to 100%, soil MBC and microbial quotient decreased, suggesting that no tillage with 60% crop residue mulching led to the strongest microorganisms assimilation—beneficial for SOC turnover.

### Effects on soil bacterial diversity and community composition

The species diversity indexes are crucial indicators of microbial community content [46]. In this study, the alpha diversity of soil bacterial community was evaluated by ACE, Chao1, Shannon, Simpson, and OTU Richness indexes. After 10 years of consecutive no tillage and residue mulching for the soybean–maize rotation system, the OTU Richness, ACE, Chao1, and Shannon indexes were higher for NTR3 than for other treatments (Fig 2); these results indicated that NTR3 can improve the availability of substrate, increase bacterial diversity and richness, and maintain high ecosystem stability. The reasons underlying these results may be that the addition of straw brings much easily usable organic matter to the soil and promotes the growth of nutrient-rich microorganisms in the soil. This improves bacterial community diversity in the soil.

Some studies have also shown that soils rich in organic carbon have lower species diversity of bacterial community [47]. In the current study, the diversity and richness indexes after NTR4 were significantly lower than those after the other treatments, possibly because residue mulching amounts for NTR4 was higher, such that most of the residue mulch was difficult to decompose within a short time and thus could not be easily used by soil microorganisms, but the easily decomposed organic carbon, in turn, lead to competition among microorganisms and thus promoted growth of certain kinds of microorganisms considerably, such that the growth of other microorganisms was retarded; as a result, there was a decrease in microbial diversity.

Soil microbial community composition is closely related to soil health, an important index for soil quality [48]. In this study, under different residue mulching methods for 10 consecutive years, soil bacterial community composition was affected significantly, where only some specific soil bacteria were enriched and domesticated.

The results of our PCoA based on weighted and unweighted UniFrac distances showed that the compositions of bacterial communities after NTR1 and NTR2 were similar, whereas those after NTR3 and NTR4 treatments varied significantly (Fig 4). Thus, no tillage with a large amount of crop residue mulching formed a nutrient-rich soil environment, which changed rhizosphere soil microbial system and significantly affected rhizosphere bacterial community composition.

The results of relative abundance analysis of bacterial taxonomic components demonstrated that the most important bacterial phylum in this study was Proteobacteria, followed by Acidobacteria and Actinobacteria—similar to previously reported results [49]. In the current study, no significant effect was noted on the dominant population types. This result may be due to the newly generated microorganisms under different residue mulching methods for 10 consecutive years not having formed a dominant flora. Here, dominant flora formation may take several decades or longer.

The relative abundance of eutrophic bacteria, such as Proteobacteria, Actinobacteria, and Bacteroidetes, were improved at different extents by NTR3 and NTR4 mulched with a large amount of residue. Alphaproteobacteria in Proteobacteria could utilize refractory carbon sources in acidic environment and degrade into small intermediate molecules to provide nutrients for other microorganisms [50]. Actinobacteria could utilize available carbon sources to grow rapidly and accelerate the turnaround and utilization of SOC [51]. The increase in the abundance of these phyla would contribute to the degradation of hard-to-decompose organic matter and the efficient utilization of carbon sources. In this research, the abundance of Acidobacteria and Verrucomicrobia was reduced in the NTR3 and NTR4 treatments. The reason maybe that, Acidobacteria and Verrucomicrobia, belonging to the oligotrophic groups [52], maintain a low growth rate in almost all environments as well as a poor ability to absorb and metabolize or degrade substances that are low in nutrients. Therefore, in soils of the treatments with rich nutrients substances, these oligotrophic microorganisms were less competitive with nutrient-rich microorganisms, subsequently resulting in a decrease in their relative abundance.

The abundance of the top 10 bacteria with low relative abundance, namely Verrucomicrobia, Patescibacteria, Planctomycetes, and Cyanobacteria, increased to varying degrees after NTR2—indicating that 30% of residue mulching in the soil environment was conducive to the growth and reproduction of bacteria with low abundance—and improved the homogeneity of the bacterial community.

### Regulation of soil traits on bacterial diversity and composition

Residue mulching or litter affects soil pH, soil moisture, soil compactness, SOC content, and other physicochemical properties [9, 53]; this results in differences in bacterial community diversity and structure in the plant rhizosphere soil [2]. In this study, we found that long-term conservation tillage using no tillage and residue mulching indirectly shaped the composition of soil bacterial communities by changing soil physicochemical properties. SOC, soil pH, and soil temperature were the main driving factors affecting soil bacterial diversity and composition (Fig 5; S6 Table), and different soil physicochemical properties had varied roles in regulating bacterial diversity and composition.

SOC, a large carbon source for soil microorganisms, and its turnover is closely related to soil bacteria community composition [54], significantly affects soil microbial community structure [55, 56]. Our results were consistent with previous work, this is because no-tillage straw mulching not only reduces the fluctuation of surface temperature, but also facilitates the accumulation of soil organic matter, which is conducive to the survival of soil microorganisms [57, 58]. Meanwhile, SOC is generally positively correlated with the activities of microbial, and soil microorganisms play an important role in soil organic matter degradation and nutrient cycling [59]. Proteobacteria and Bacteroidetes are considered copiotrophs, which prefer a high-nutrient environment and maintain a high nutrient availability when the soil environment is conducive to the growth of soil microorganisms [60, 61]. Li et al. [62] indicated that the relative abundance of Actinobacteria is closely related to increases in carbon source content. Yu et al. [63] found that after 6 continuous years of residue mulching, SOC content in the soil increased, the relative abundance of Chloroflexi in the soil was significantly higher than that residue removal treatment. These results corroborate the current results. The residue mulching significantly increased SOC, providing carbon source substrate for the bacterial growth and reproduction. SOC had a significant positive correlation with the relative abundance of Proteobacteria, Actinobacteria, Bacteroidetes, and Chloroflexi.

Soil pH has been identified as a major contributor to microbial diversity and community composition in many studies [64]. This is because many soil characteristics, including soil nutrient availability, ion concentration in soil solution, and organic carbon properties, are often directly or indirectly related to soil pH [65], and these factors may result in changing of soil microbial composition. Another reason maybe that soil pH directly affects the habitat of soil microorganisms, thus changing the composition of soil microbial community [66]. In the current study, the relative abundance of Gemmatimonadetes, Cyanobacteria, and Acidobacteria was significantly correlated with pH. Cederlund et al. [67] reported that the adaptability of Gemmatimonadetes to the soil environment was related to the carbon and nitrogen environment, and the relative abundance of Gemmatimonadetes increased significantly after a residue mulching treatment [2], possibly because residue returning changed the environment of soil carbon and nitrogen, which indirectly affected soil pH. This drove the change of relative abundance of Gemmatimonadetes. Most studies have reported that the differences in the relative abundance of Acidobacteria was attributable to soil pH differences; moreover, the relative abundance of Acidobacteria was significantly and negatively correlated with soil pH [68, 69]. In the current study, Acidobacteria abundance was significantly and positively correlated with soil pH, which was consistent with the results of the research on sweet potato continuous cropping [70] and the study on diversity of bacterial community in cotton field [71]. This result may be related to the difference in response to soil environmental factors between different Acidobacteria subgroups or even between Acidobacteria in the same subgroup [68, 72]. In terms of rhizosphere potency, compared with non-rhizosphere soil, the pH of rhizosphere soil is lower (organic acid secreted), which is conducive to the growth of some subgroups of Acidobacteria. However, the high-nutrient environment in the rhizosphere is not conducive to the colonization and reproduction of Acidobacteria. The distribution of Acidobacteria in plant rhizosphere results from the comprehensive action of various factors in the rhizosphere microenvironment. Therefore, the relationship between Acidobacteria in rhizosphere soil and soil pH cannot be said sweepingly.

The changes in soil temperature could directly affect the growth, mineralization rate, enzyme activity and community composition of soil microorganisms [73]. Meanwhile, the increasing of soil temperature could indirectly affect soil microbial community through affecting the primary productivity of plant, the carbon input underground, soil water and nutrient availability. In this study, most bacterial phyla were significantly and positively correlated with soil temperature, while the relative abundance of Proteobacteria, Actinobacteria, and Bacteroidetes had a significant negative correlation with it. The reason here was that straw mulching increased SOC storage by reducing the fluctuation of soil temperature and water content, which was beneficial to improve the abundance of eutrophic bacteria.

Taken together, our results indicated that changes in soil physicochemical properties due to long-term different residue mulching methods represented an important reason for differences in soil microbial structural diversity.

## Conclusions

The diversity and composition of the soil bacterial community in residue mulching soil were significantly different from those in residue removal soil after 10 years of no tillage in a black soil region of Northeastern China. Residue mulching method showed indirect quantitative effects on diversity and composition of bacteria by altering SOC, soil pH, and soil temperature. Soil bacterial richness and diversity were improved after no tillage with 60% residue mulching, demonstrating potential to improve soil health, and this was the most suitable residue mulching quantity in the black soil area of Sanjiang Plain. We suggest strip tillage is more suitable for 100% residue returned to the field in the cold region of northeast China.

Our findings enhance the understanding of the role of no tillage and residue mulching in maintaining soil fertility and altering soil bacterial community in cold regions. The current findings provide a scientific basis for the optimization of no tillage measures in the black soil area of Sanjiang Plain in Northeastern China.

## Supporting information

S1 Table Detailed sequencing depth results of soil samples after different no tillage and residue mulching treatments. (DOCX)

S2 Table Statistics of tags among soil samples after different no tillage and residue mulching treatments. (DOCX)

S3 Table The diversity and richness of soil bacterial according to the Chao1, ACE, Shannon, and Simpson indexes after different no tillage and residue mulching treatments. (DOCX)

S4 Table Relative abundance of top 10 soil bacterial phyla for all samples after different no tillage and residue mulching treatments. (DOCX)

S5 Table Explanatory variable redundancy analysis for soil bacterial community composition. (DOCX)

S6 Table Contribution and significance of environmental variables to soil bacterial community composition. (DOCX)
