# Supplementary material for: No tillage and residue mulching method on bacterial community diversity regulation in a black soil region of Northeastern China
Source: PLoS One. 2021 Sep 10;16(9):e0256970. doi: 10.1371/journal.pone.0256970 (PMC8432829; doi:10.1371/journal.pone.0256970)
Supplement: S1 Table — (DOCX) [file pone.0256970.s001.docx]

**S1 Table. Detailed sequencing depth results of soil samples after different no tillage and residue mulching treatments.**

| Sample | PE_reads | Clean_tags | Effective_tags | OTU_conts | Goods_coverage |
| --- | --- | --- | --- | --- | --- |
| NTR11 | 79943 | 77301 | 72617 | 1358 | 0.997807 |
| NTR12 | 76707 | 73213 | 67652 | 1342 | 0.996601 |
| NTR13 | 51881 | 49737 | 46833 | 1323 | 0.995005 |
| NTR21 | 80073 | 76471 | 71519 | 1390 | 0.997951 |
| NTR22 | 80002 | 76473 | 68827 | 1351 | 0.995193 |
| NTR23 | 63476 | 60345 | 53948 | 1299 | 0.992277 |
| NTR31 | 54093 | 51548 | 46819 | 1333 | 0.991693 |
| NTR32 | 79996 | 76751 | 70756 | 1400 | 0.998678 |
| NTR33 | 79978 | 77875 | 71576 | 1397 | 0.998197 |
| NTR41 | 79862 | 77159 | 72481 | 1289 | 0.997317 |
| NTR42 | 48671 | 46903 | 43813 | 1286 | 0.993521 |
| NTR43 | 79894 | 76888 | 71002 | 1359 | 0.997302 |
| Total | 854576 | 820664 | 757843 | 1438 | - |

NTR1, no tillage and all crop residue removed; NTR2, no tillage and 30% crop residue mulching; NTR3, no tillage and 60% crop residue mulching; NTR4, no tillage and all crop residue mulching. The numbers following the treatment name denote the sampling replications. For example, NTR11, NTR12, and NTR13 means the soil sampling was taken from replicate 1, 2, and 3 of the field plots, respectively.
