# Supplementary material for: No tillage and residue mulching method on bacterial community diversity regulation in a black soil region of Northeastern China
Source: PLoS One. 2021 Sep 10;16(9):e0256970. doi: 10.1371/journal.pone.0256970 (PMC8432829; doi:10.1371/journal.pone.0256970)
Supplement: S5 Table — (DOCX) [file pone.0256970.s005.docx]

**S5 Table. Explanatory variable redundancy analysis for soil bacterial community composition**

| Item | Eigenvalues | Explained variation (cumulative) % | Pseudo-canonical correlation | Explained fitted variation (cumulative) % |
| --- | --- | --- | --- | --- |
| Axis1 | 0.5229 | 52.29 | 0.9187 | 66.70 |
| Axis2 | 0.1648 | 68.77 | 0.977 | 88.11 |
| Axis3 | 0.0668 | 75.45 | 0.8081 | 96.67 |
| Axis4 | 0.0259 | 78.05 | 0.9666 | 100.00 |
