# Supplementary material for: No tillage and residue mulching method on bacterial community diversity regulation in a black soil region of Northeastern China
Source: PLoS One. 2021 Sep 10;16(9):e0256970. doi: 10.1371/journal.pone.0256970 (PMC8432829; doi:10.1371/journal.pone.0256970)
Supplement: S6 Table — (DOCX) [file pone.0256970.s006.docx]

**S6 Table. Contribution and significance of environmental variables to soil bacterial community composition.**

| Soil property | Explains(%) | F | P |
| --- | --- | --- | --- |
| SOC | 22.3 | 2.9 | 0.068 |
| pH | 21.3 | 2.7 | 0.081 |
| Temperature | 18.3 | 2.2 | 0.098 |
| TK | 18.3 | 2.2 | 0.104 |
| BD | 12.0 | 1.4 | 0.264 |
| TN | 9.2 | 1.0 | 0.352 |
| Moisture | 6.7 | 0.7 | 0.547 |
| Tp | 4.1 | 0.4 | 0.754 |

BD, soil bulk density; SOC, soil organic carbon; TN, total nitrogen; TP, total phosphorus; TK, total potassium
